# Validity of a reported history of chickenpox in targeting varicella vaccination at susceptible adolescents in England^☆^

**DOI:** 10.1016/j.vaccine.2013.06.098

**Published:** 2014-02-26

**Authors:** Nigel Field, Gayatri Amirthalingam, Pauline Waight, Nick Andrews, Shamez N. Ladhani, Albert Jan van Hoek, Peter A.C. Maple, Kevin E. Brown, Elizabeth Miller

**Affiliations:** a Immunisation, Hepatitis and Blood Safety Department, Health Protection Services, Health Protection Agency, 61 Colindale Avenue, London NW9 5EQ, United Kingdom^1^; b Research Department of Infection & Population Health, University College London, Mortimer Market Centre, Off Capper Street, London WC1E 6JB, United Kingdom; c Statistics Modelling and Economics Department, Health Protection Services, Health Protection Agency, 61 Colindale Avenue, London NW9 5EQ, United Kingdom^1^; d Virus Reference Department, HPA Microbiology Services, Health Protection Agency, 61 Colindale Avenue, London NW9 5EQ, United Kingdom^1^

**Keywords:** Varicella, Chickenpox, Reported history, Validity, Adolescent, Vaccination programme

## Abstract

**Introduction:**

In the UK, primary varicella is usually a mild infection in children, but can cause serious illness in susceptible pregnant women and adults. The UK Joint Committee on Vaccination and Immunisation is considering an adolescent varicella vaccination programme. Cost-effectiveness depends upon identifying susceptibles and minimising vaccine wastage, and chickenpox history is one method to screen for eligibility. To inform this approach, we estimated the proportion of adolescents with varicella antibodies by reported chickenpox history.

**Methods:**

Recruitment occurred through secondary schools in England from February to September 2012. Parents were asked about their child's history of chickenpox, explicitly setting the context in terms of the implications for vaccination. 247 adolescents, whose parents reported positive (120), negative (77) or uncertain (50) chickenpox history provided oral fluid for varicella zoster virus-specific immunoglobulin-G (VZV-IgG) testing.

**Results:**

109 (90.8% [85.6–96.0%]) adolescents with a positive chickenpox history, 52 (67.5% [57.0–78.1%]) with a negative history and 42 (84.0% [73.7–94.3%]) with an uncertain history had VZV-IgG suggesting prior infection. Combining negative and uncertain histories, 74% had VZV-IgG (best-case). When discounting low total-IgG samples and counting equivocals as positive (worst-case), 84% had VZV-IgG. We also modelled outcomes by varying the negative predictive value (NPV) for the antibody assay, and found 74–87% under the best-case and 84–92% under the worst-case scenario would receive vaccine unnecessarily as NPV falls to 50%.

**Conclusion:**

Reported chickenpox history discriminates between varicella immunity and susceptibility in adolescents, but significant vaccine wastage would occur if this approach alone were used to determine vaccine eligibility. A small but important proportion of those with positive chickenpox history would remain susceptible. These data are needed to determine whether reported history, with or without oral fluid testing in those with negative and uncertain history, is sufficiently discriminatory to underpin a cost-effective adolescent varicella vaccination programme.

## Highlights

• Chickenpox history may enable cost-effective vaccination of susceptible individuals.
• We tested the validity of reported chickenpox history in adolescents.
• Vaccine would be wasted in most adolescents with a negative or uncertain history.
• 6–9% of those with a positive chickenpox history would remain susceptible.
• These data are needed to inform cost-effectiveness of proposed vaccine programmes.

## 1 Introduction

Primary varicella infection (chickenpox) is an acute illness caused by varicella-zoster virus (VZV), which is characterised by a generalised vesicular rash, fever and malaise. [1] In the UK, most chickenpox occurs in children under 10 years old and is mild. Seroprevalence data suggest 80% of 11-year-olds in England and Wales have previously been exposed to varicella infection. [2] Serious illness mainly occurs in immunocompromised individuals and the remaining susceptible adults, which is of particular concern in pregnancy, and may lead to maternal complications (e.g. varicella pneumonia) and severe foetal consequences (e.g. congenital varicella syndrome). When VZV reactivates from dorsal root ganglia in later life, this causes a painful dermatomal rash known as herpes zoster or shingles.

Universal varicella immunisation has not been introduced in the UK, in part due to concerns that this may shift the burden of primary disease to susceptible adults, who are at higher risk of complications, [3–5] and increase shingles reactivations, due to reduced natural boosting in those previously exposed [4,5]. A recent review by the Joint Committee on Vaccination and Immunisation (JCVI) concluded that a two-dose childhood varicella vaccination schedule was not cost-effective, but JCVI did recommend a single-dose herpes zoster vaccine for adults aged 70–79 [6].

To prevent severe primary infections among adults, alternative approaches to routine childhood varicella vaccination are being considered in the UK [7]. In particular, the introduction of a selective adolescent varicella vaccination programme may be cost-effective [5]. Given that most adolescents will have acquired natural immunity, the cost-effectiveness of this approach will largely depend upon accurate pre-immunisation identification of susceptibles to minimise vaccine wastage in those already immune. Two screening methods are available: reported chickenpox history, or laboratory testing for VZV-specific immunoglobulin G (IgG) antibody, which is significantly more expensive, more time consuming and likely to involve higher dropout rates.

Understanding the validity of reported chickenpox history in the target group is essential to inform this decision, and to model the impact and cost-effectiveness of the overall approach. Oral fluid (gingivocrevicular fluid) is simple and non-invasive to collect, and with appropriately sensitive assays can be used for the detection of viral antibodies for seroprevlance studies [8]. This study estimates the proportions of adolescents already immune to VZV, by reported chickenpox history, using detection of VZV-specific antibodies in oral fluid as a serological correlate suggesting previous infection.

## 2 Methods

Recruitment occurred during February to September 2012. The study aimed to recruit a group broadly representative of the British general population, where approximately 15% of adolescents are of non-white ethnicity, [9] because differences in the predictive value of chickenpox history by ethnicity have been reported. [10,11] Adolescents were therefore recruited through two secondary schools in South London to increase the number of non-white participants, and two other regions of England (Hertfordshire and Gloucestershire). Participating schools provided all students aged 11–15 with study information packs to take home to their parents. Individuals with any serious health condition causing immune dysfunction, who would be ineligible to receive a live vaccine, and those who had previously received a varicella vaccine were excluded.

Study packs asked parents to return a short questionnaire by post, including their child's ethnicity and the following question about chickenpox history: “*Most children will have had chickenpox by the time they are 10 years old. Chickenpox infection provides long-term protection against future infection and there is no need for vaccination if someone has already had chickenpox. We want you to think about your child's history of chickenpox in this context. Has your child had chickenpox?”* Answers were: (1) “*Yes (If yes, your child does not need chickenpox vaccine)”*, (2) “*No”* or (3) “*I don’t know”.* To increase the number of negative and uncertain responses towards the end of the study, after receiving over 500 positive responses, the question was altered to, “*Has your child had chickenpox?* (*If you answer yes, we do NOT need you to complete or return this form as we have now received enough replies from this group*)*”*.

Parents who returned the questionnaire were sent a consent form and a kit to collect oral fluid, with clear instructions on how to obtain a sample from their child, which they were asked to return to the Health Protection Agency (HPA). Approximately 7000 introductory letters were distributed by schools; 550 questionnaires were returned with a positive history of chickenpox, 84 with a negative history, and 56 with an uncertain history, and 1 was incomplete. We posted 268 oral fluid kits, including 128 to respondents with a positive history of chickenpox and all those with negative or uncertain histories.

Families were informed at the outset in the initial study information pack that, as a token of appreciation, a voucher for £10 would be sent to them once a sample was received in the laboratory. Children found to be susceptible to varicella were offered two doses of varicella vaccine without charge.

### 2.1 Laboratory procedures

Oral fluid samples and consent forms were received by the HPA Virus Reference Department, MS-Colindale, and processed to extract VZV-IgG using standard methods and diluents. Oral fluid samples were stored at −30 °C prior to batch testing. For semi-quantitative determination of IgG antibodies to VZV, the in-house VZV-IgG time resolved fluorescence immunoassay, (TRFIA), [12] was modified for testing oral fluid. Testing of paired serum and oral fluid samples, had previously established that measurements above a cut-off of 0.35 mIU/mL should be considered positive, below a cut-off of 0.25 mIU/mL as negative, with an equivocal range between 0.25 and 0.35 mIU/mL. [HPA unpublished data] Samples testing negative or equivocal were also tested for total IgG to determine whether the sample had been taken appropriately and contained sufficient total IgG, using a cut-off of greater than 2.5 mg/L.

### 2.2 Data analysis

Data were analysed using Stata v12 (Statcorp, TX, US). For each chickenpox history group, we aimed for a sample size of 100, to estimate with reasonable precision the proportion with VZV-IgG (95% confidence interval within ±10%). The study was not designed or powered to detect differences by ethnicity. Exact 95% confidence intervals for proportions were calculated and proportions compared according to history using two-sided Fisher's exact tests. We also undertook a sensitivity analysis to investigate the impact of using the oral fluid assay in populations with different VZV-IgG prevalence by modelling the effect of different values for the negative predictive value (NPV) of the assay.

## 3 Results

120 oral fluid samples were received from respondents with a positive history of chickenpox, 77 with a negative history and 50 with an uncertain history. The average age of respondents was 13 years, and 85% were white, 6% mixed ethnicity, 6% Asian, 3% Black, and 1% Chinese. The groups with different history responses were not significantly different with respect to age or ethnicity (data not shown).

Overall, 109 (90.8% [95% CI 85.6–96.0%]) with a positive history of chickenpox, 52 (67.5% [57.0–78.1%]) with a negative history and 42 (84.0% [73.7–94.3%]) with an uncertain history had VZV-IgG antibodies indicating previous varicella infection (Table 1). 16 oral fluid samples were found to have insufficient total IgG for reliable detection of specific VZV-IgG, including 13 (81%) from respondents with a negative or uncertain history, suggesting these may be true negatives. To assess the best-case scenario, our initial analysis therefore grouped together negative, equivocal, and insufficient oral fluid results (Table 2). Under these conditions, 11 (9.1% [4.0–14.4%]) with a positive history, 25 (32.5% [21.2–43.0%]) with a negative history and 8 (16.0% [5.7–26.3%]) with an uncertain history had no evidence of previous varicella infection. An adolescent varicella immunisation programme would offer the vaccine to those with either a negative or uncertain history, of whom 94 (74.0% [66.3–81.7%]) were positive for VZV-IgG and 33 (26.0% [18.3–33.7%]) were negative.

To assess the worst-case scenario, our second analysis discounted samples with insufficient IgG and assumed equivocal results were positive (Table 3). Under these conditions, 96 (84.2% [77.5–91.0%]) with a negative or uncertain history of chickenpox had antibodies indicating previous varicella infection.

**Fig. 1:**
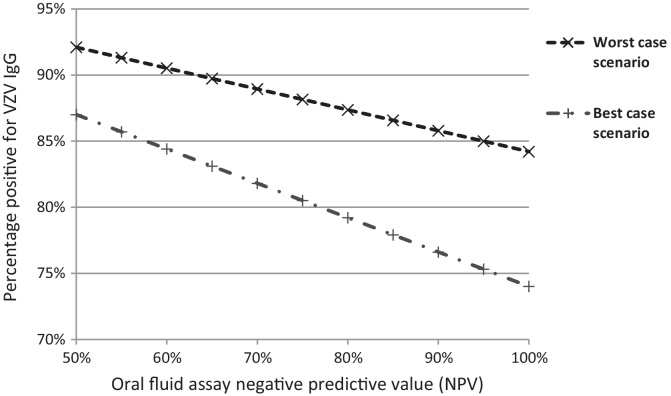
The effect of varying the negative predictive value (NPV) of the oral fluid assay on the proportion of individuals with negative or uncertain chickenpox history and evidence of previous varicella infection.

Using paired serum and oral fluid samples, the assay used in this study was previously shown to have a sensitivity of 96.3% and specificity of 90.9%. [HPA unpublished data] In populations with a high seroprevalence of VZV-IgG, the positive predictive value (PPV) of this assay will approach 100%, but NPV may be lower. To explore this, we assumed the PPV to be 100% and varied the NPV between 50% and 100%. Using the study data as described above, Fig. 1 shows the impact on the expected proportion of respondents with a negative or uncertain chickenpox history testing positive for VZV-IgG (i.e. the proportion of vaccine-eligible individuals who might receive vaccine unnecessarily). Under the best-case scenario, this proportion increased from 74% to 87% and under the worst-case scenario from 84% to 92% as NPV falls to 50%.

## 4 Discussion

Adolescent varicella vaccination is being considered in the UK with the aim of preventing serious adult disease and to avoid infection in pregnancy in those susceptible. Previous reviews have found antenatal screening for varicella, and childhood vaccination not to be cost-effective [6,13]. Cost-effectiveness of an adolescent varicella vaccination programme in the UK is likely to depend on the proportion of vaccine doses given unnecessarily to individuals with prior natural immunity. We therefore assessed the validity of reported chickenpox history to determine vaccine eligibility, by asking parents about their child's history of chickenpox, explicitly setting the context in terms of the implications for vaccination. We then tested the adolescents for varicella antibodies to determine previous exposure. At best, 68% of those with negative history and 74% of those with a negative or uncertain history might receive vaccine unnecessarily. At worst, vaccine would be wasted in 81% of those with negative history and 84% with negative or uncertain history. These data provide a useful range of estimates to model the likely cost-effectiveness of preventing adult varicella disease by vaccinating adolescents.

We also provide estimates for the proportion of adolescents with a positive history of chickenpox and no evidence of previous varicella infection (6–9%), who would remain susceptible if disease history was used to determine vaccine eligibility. This group may comprise a substantial proportion of all susceptibles in the population because the majority of the population is likely to have a positive history. These data will inform modelling estimates of the remaining disease burden following implementation of a vaccine programme based on chickenpox history. Cost-effectiveness analysis would also take account of immunocompromised susceptibles, who would not be eligible for a live attenuated vaccine but would be at greater risk of severe disease.

Other countries have adopted adolescent varicella immunisation strategies, including Australia, where a school-based immunisation programme targeting adolescents aged 10–13 years with no previous history of chickenpox or varicella vaccination has been in place since 2006 [14], and European countries such as Austria, Cyprus, Germany, Greece, Italy, Spain and Turkey [15]. Some previous studies have investigated the validity of chickenpox history in adolescents, for example, in Greece [16], Switzerland [17], Turkey [18], and the American military [19]. Other studies have investigated other groups at other ages, for example, health care workers [11,20,21], hospital patients, [22,23] pregnant women [24–26], refugees [27], and army recruits [28,29]. Many studies are set in other countries, where the natural history and prevalence of varicella infection differs, and sometimes with different objectives, such as to decide the risk in pregnant women following exposure to chickenpox infection [30], where the tolerance for error is much lower. As such, there is a broad range of published estimates for the proportion of individuals with negative or uncertain chickenpox history and previous varicella infection [32,33], and in some cases this is extremely low (11%) [31], which makes generalisation difficult. Our study is the first, to the best of our knowledge, to frame the history question about previous chickenpox disease specifically within the context of the implications for vaccination of adolescents.

To maximise the use of our data in other settings, where the prevalence of varicella (and therefore NPV) may differ, we present all assay data from the study (Table 1), including equivocal results and samples with low total IgG, and model the impact of varying the assay NPV between 50% and 100% (Fig. 1). The oral fluid assay, using a modified TRFIA to detect specific VZV-IgG antibody, was chosen because it avoids any invasive procedure to collect blood and is more likely to be acceptable to parents and adolescents, thus improving study response rates.

A recently proposed change to the UK adolescent vaccination programme would mean that a group C meningococcal booster vaccine may be offered with the Td/IPV (tetanus, diphtheria, polio) booster to those aged 13–14 [34], and an adolescent varicella vaccination programme could be given at the same time. The average age of participants in this study was 13 years, and the study population intentionally reflects ethnic diversity in the UK adolescent general population through the inclusion of two schools in South London to increase the number of non-white respondents. Among all study respondents providing an oral fluid sample, 82% tested positive for VZV-IgG, which reflects the likely prevalence in the UK for this age group. [2] Our study, however, did not aim to provide population prevalence estimates for the different chickenpox history responses because it was not possible to assess how accurately respondents reflect the population. For example, parents of adolescents with negative or uncertain histories may have been more likely to participate given the provision of free vaccine to those without VZV-IgG antibodies. The proportion with different histories may also have been affected by changing the question about chickenpox history at the end of the study to boost the number of negative and uncertain responses, and the small token of appreciation offered. Finally, it is difficult to foresee how parents’ answers might be influenced by the prospect of their child actually receiving a vaccine in the context of a national adolescent vaccination programme.

## 5 Conclusion

We show that asking parents to report their child's chickenpox history can significantly discriminate between adolescents who are immune and susceptible to varicella infection. These data will be used to determine by modelling whether reported history, with or without oral fluid testing in those with negative or uncertain history, is sufficiently discriminatory to underpin a cost-effective varicella vaccination programme that will protect susceptibles against chickenpox in the UK.

## Ethical approval

Ethical approval was granted by the London Harrow National Research Ethics Service (11/LO/1916).

## Funding

The field and laboratory work for this study were supported by a grant from the DH Research and Development Directorate, grant number 039/0031. The views expressed in the publication are those of the authors and not necessarily those of the Department of Health, England. Nigel Field is supported by a NIHR Academic Clinical Lectureship. The funding sources had no role in data collection, data analysis, data interpretation or writing of the report.

## Authors’ contributions

The study was designed and implemented by NF, GA, PW, NA, AJvH, KEB and EM, with EM as the Chief Investigator. NF wrote the first draft of the paper and did the analysis, with significant contributions from NA. PW assisted with the study fieldwork, participant follow-up and data management, with contributions from GA and SNL. KEB designed and coordinated laboratory testing, which was undertaken by CPM. AJvH advised on the use of study data for cost-effectiveness modelling. All investigators contributed to and approved the final version of the paper.

## Figures and Tables

**Table 1:** Reported history of chickenpox and VZV-IgG results.

	VZV IgG Result				Total
	Positive	Negative	Equivocal	Insufficient	
	*n* (%)	*n* (%)	*n* (%)	*n* (%)	*n*
Chickenpox history					
Positive	109 (90.8%)	7 (5.8%)	1 (0.8%)	3 (2.5%)	120
Negative	52 (67.5%)	13 (16.9%)	2 (2.6%)	10 (13.0%)	77
Uncertain	42 (84.0%)	5 (10.0%)	0 (0.0%)	3 (6.0%)	50
Total	203 (82.2%)	25 (10.1%)	3 (1.2%)	16 (6.5%)	247

**Table 2:** Best-case scenario: validity of chickenpox history, grouping together negative, equivocal and insufficient IgG.

	VZV IgG Result				Total
	Positive		Negative, equivocal or insufficient		
	*n*	% [95% CI]	*n*	% [95% CI]	*n*
Chickenpox history					
Positive	109	90.8% [85.6–96.0%]	11	9.1% [4.0–14.4%]	120
Negative	52	67.5% [57.0–78.1%]	25	32.5% [21.2–43.0%]	77
Uncertain	42	84.0% [73.7–94.3%]	8	16.0% [5.7–26.3%]	50
Negative or uncertain	94	74.0% [66.3–81.7%]	33	26.0% [18.3–33.7%]	127

p-Values for comparison of proportions were calculated as follows: positive vs. negative: *p* < 0.001; positive vs. negative or uncertain: *p* < 0.001; positive vs. uncertain: *p* = 0.284; negative vs. uncertain: *p* = 0.041.

**Table 3:** Worst-case scenario: validity of chickenpox history, discounting insufficient IgG and counting equivocal as positive.

	VZV IgG Result				Total
	Positive or equivocal		Negative		
	*n*	% [95% CI]	*n*	% [95% CI]	*n*
Chickenpox history					
Positive	110	94.0% [89.7–98.4%]	7	6.0% [1.6–10.3%]	117
Negative	54	80.6% [71.0–90.2%]	13	19.4% [9.8–29.0%]	67
Uncertain	42	89.4% [80.4–98.3%]	5	10.6% [1.7–20.0%]	47
Negative or uncertain	96	84.2% [77.5–91.0%]	18	15.8% [9.0–22.5%]	114

*p*-Values for comparison of proportions were calculated as follows: positive vs. negative: *p* = 0.007; positive vs. negative or uncertain: *p* = 0.020; positive vs. uncertain: *p* = 0.327; negative vs. uncertain: *p* = 0.297.
